# Evaluation of Radiation Dose and Image Quality in the Transition from Conventional Pelvimetry to Low-Dose Helical CT Pelvimetry

**DOI:** 10.3390/tomography12030035

**Published:** 2026-03-04

**Authors:** K. Shahgeldi, M. Parenmark, L. Claesson, T. M. Svahn

**Affiliations:** 1 Department of Radiation Therapy, Uppsala University Hospital, 751 85 Uppsala, Sweden; 2 Department of Imaging and Functional Medicine, Division Diagnostics, Gävle Hospital, Region Gävleborg, 801 88 Gävle, Sweden; 3 Centre for Research and Development, Uppsala University, Region Gävleborg, 801 88 Gävle, Sweden

**Keywords:** pelvimetry, low-dose CT, radiation dose, fetal dose, effective dose, Monte Carlo simulation, image quality, obstetric imaging

## Abstract

Purpose: The present study aimed to assess the radiation dose associated with low-dose (LD) CT pelvimetry compared with conventional radiography and to evaluate the adequacy of the resulting image quality. Methods: The absorbed dose was measured using thermoluminescent dosimeters positioned in an anthropomorphic female phantom, including uterine locations, to estimate the fetal dose. Conventional radiographic pelvimetry and LD-CT pelvimetry were performed using clinically implemented protocols. Effective dose was calculated using Monte Carlo–based modeling applying acquisition parameters and retrospective patient dose registry data. Image quality of LD-CT pelvimetry was independently evaluated in 14 consecutive clinical cases using a four-point ordinal scale. Results: LD-CT pelvimetry reduced the mean absorbed pelvic dose by approximately 50% compared with conventional pelvimetry (0.18 vs. 0.39 mGy) and decreased estimated fetal dose by 40% (0.21 vs. 0.37 mGy). These estimates were based on standardized single acquisitions and did not incorporate additional radiation from retakes commonly observed in conventional practice. CT demonstrated substantially more homogeneous dose distribution, whereas conventional pelvimetry exhibited marked heterogeneity with peak values up to 2.3 mGy. The maternal effective dose was lower for LD-CT (0.16 mSv) than for conventional pelvimetry (0.36 mSv); inclusion of retakes increased the conventional effective dose to 0.71 mSv. All CT examinations were diagnostically adequate, and no recalls were required. Conclusions: Optimized low-dose CT pelvimetry significantly reduces radiation dose compared with conventional radiographic pelvimetry while maintaining reliable diagnostic image quality. These results support the clinical adoption of CT-based pelvimetry as a dose-efficient and reproducible alternative to conventional techniques.

## Simple Summary

Conventional radiographic pelvimetry remains in clinical use to assess pelvic dimensions prior to delivery; however, it often requires multiple projections and may be associated with relatively high radiation doses. As part of a planned ten-year equipment renewal cycle, we evaluated low-dose CT as an alternative approach. Using phantom measurements and patient dose data, we found that low-dose CT pelvimetry reduced pelvic and estimated fetal radiation dose by approximately 40–75% compared with conventional radiography, particularly when accounting for image retakes. Image quality was diagnostically sufficient in all CT examinations, and no repeat scans were required. These findings support the implementation of optimized low-dose CT as a safer, more reproducible, and dose-efficient method for pelvimetric assessment.

## 1. Introduction

Pelvimetry is a diagnostic examination used to assess pelvic dimensions in women for whom vaginal delivery may be challenging, to identify an increased risk of obstructed labor, and to determine the potential need for cesarean section [1]. Although clinical pelvimetry remains central to obstetric decision-making, imaging-based pelvimetry may be indicated when clinical findings suggest a disproportion between the fetal head and the maternal pelvis or when previous obstetric complications raise concern [2,3]. Accurate assessment of pelvic dimensions may help reduce the risk of emergency cesarean delivery, which is associated with higher maternal morbidity than planned procedures [4].

Conventional radiographic pelvimetry has historically been the most widely used imaging technique for this purpose. Several studies have shown that radiographic pelvimetry can provide reliable measurements of pelvic dimensions relevant to obstetric management [3,5]. However, this technique requires multiple two-dimensional projections, typically frontal and lateral views, which may result in relatively high radiation exposure, particularly when image retakes are necessary. Retakes are common due to positioning challenges, geometric constraints, or inadequate visualization of key anatomical landmarks, and they can substantially increase patient and fetal radiation dose.

Alternative imaging modalities have been investigated to reduce radiation exposure. Magnetic resonance imaging (MRI) provides a three-dimensional assessment of the pelvis without ionizing radiation and demonstrates satisfactory measurement reproducibility [6]. However, MRI is time-consuming, costly, and not universally available, particularly in acute or resource-limited settings. Ultrasonography offers wide availability and portability but currently provides limited accuracy and reproducibility for pelvimetric measurements, especially for deeper bony structures [7,8]. As a result, radiographic pelvimetry remains in clinical use in many institutions.

Computed tomography (CT) pelvimetry provides three-dimensional, geometrically accurate visualization of pelvic anatomy and has been shown to reduce interobserver variability compared with conventional techniques [9,10,11]. Historically, concerns about radiation dose have limited its widespread adoption, particularly in pregnant patients, because the scan field includes the fetus and radiosensitive maternal organs [12,13]. Fetal radiation exposure is of particular concern, as stochastic radiation effects depend on gestational age and absorbed dose, and careful justification and optimization of imaging examinations during pregnancy are essential [12,13,14,15,16].

Over the past two decades, substantial advances in CT hardware and reconstruction techniques have enabled significant reductions in radiation dose. Iterative reconstruction methods, including model-based and artificial intelligence-assisted approaches, maintain acceptable image quality at substantially lower doses [17,18]. More recently, photon-counting CT systems have been introduced and are expected to improve dose efficiency and image quality further [4,9,19]. These developments have enabled ultra-low-dose CT protocols that approach or even undercut the radiation levels of conventional radiographic examinations for selected clinical indications [20].

The present study was initiated following the planned phase-out of conventional computed radiography pelvimetry systems as part of a routine equipment renewal cycle, providing an opportunity to evaluate low-dose CT pelvimetry as a potential alternative. This study aimed to (i) compare radiation doses, including the estimated fetal dose, between low-dose CT and conventional pelvimetry using phantom measurements and patient dose data, and (ii) assess whether image quality with the low-dose CT protocol is sufficient for clinical pelvimetric measurements.

## 2. Materials and Methods

### 2.1. Phantom Model

**Figure 1:**
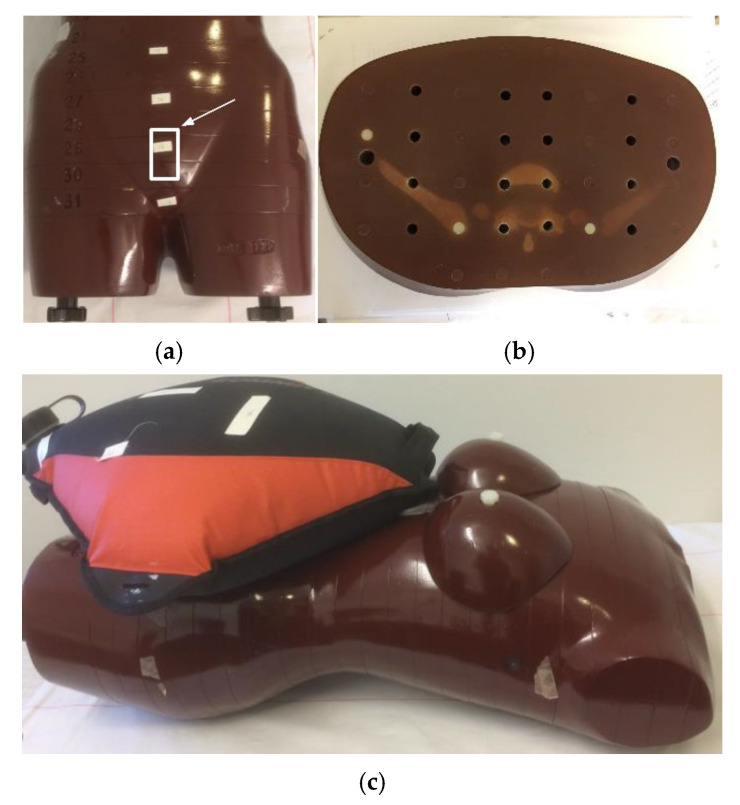
(**a**) The Alderson Radiation Therapy (ART) female phantom (the arrow indicates TLD placement for measuring the dose to the uterus); (**b**) slab No. 26 with 16 holes for TLD insertions; and (**c**) the ART phantom with the water container (10 L) simulating a 60 kg pregnant female.

This study used the female Alderson phantom (Alderson Rando Phantom, ART Phantom; The Phantom Laboratory, Salem, NY, USA), a commercially available model representing the female human body. The phantom is constructed from tissue-equivalent materials that meet the standards specified in the International Commission on Radiation Units and Measurements Report 44 (ICRU-44). These materials mimic the attenuation properties and densities of human tissues. For example, typical mass densities are 0.99, 0.32, 1.08, and 1.17 g/cm^3^ for soft tissue, lung tissue, cortical bone, and trabecular bone, respectively. The design includes transverse slices (Figure 1a), each 2.5 cm thick, with holes for inserting dosimeters. This setup enables accurate measurement of radiation dose distribution across organs, supporting quality assurance and research in medical imaging. The uterine dose is commonly used as a surrogate for estimating fetal dose [21]. The uterine measurement position is shown in Figure 1a (above the pelvis and below the water container). The original phantom is 155 cm tall and weighs 50 kg, corresponding to body mass index (BMI) of 20.8 kg/m^2^. To simulate a pregnant female weighing about 60 kg (BMI 25 kg/m^2^), a 10 L water container was placed on the phantom’s abdomen (Figure 1b). A weight gain of approximately 10 kg is typical for a woman of normal BMI (18.5–24.9) up to the 36th week of pregnancy [22].

### 2.2. Measurements of Radiation Dose to the Pelvic Region

To determine the radiation dose delivered by each imaging technique, the absorbed dose was measured directly with thermoluminescence dosimeters (TLDs, MCP-N type; LiF: Mg, Cu, P) placed in the Alderson phantom. The TLDs were calibrated according to international protocols and positioned within an acrylic calibration phantom with a 10 mm buildup layer to account for scattered radiation, as previously described [23,24]. Calibration was performed under reproducible reference conditions using X-ray beams across the study energy range (60–125 kVp), and dose measurements were obtained with a Piranha dose meter (R100; RTI Group, Mölndal, Sweden). Before study initiation, the Piranha dose meter was calibrated by the manufacturer in accordance with national standards [23].

Scout image doses were included in the CT scan measurements. The TLDs were placed in six slabs within the pelvic region (14 TLDs per slab) to cover the entire pelvis (slab numbers 26–31, Table 1). In addition, five TLDs were positioned at the uterine location to estimate the fetal dose (Figure 1a,b) [21]. Each TLD was numbered and positioned identically during both CT and conventional pelvimetry measurements to ensure consistency. The average, maximum, and standard deviation (SD) of absorbed doses were calculated. Dose distribution from individual TLD measurements was plotted using MATLAB (v. 9.0.0).

### 2.3. Image Acquisition

#### 2.3.1. Conventional Pelvimetry Imaging Procedure/Protocol

**Figure 2:**
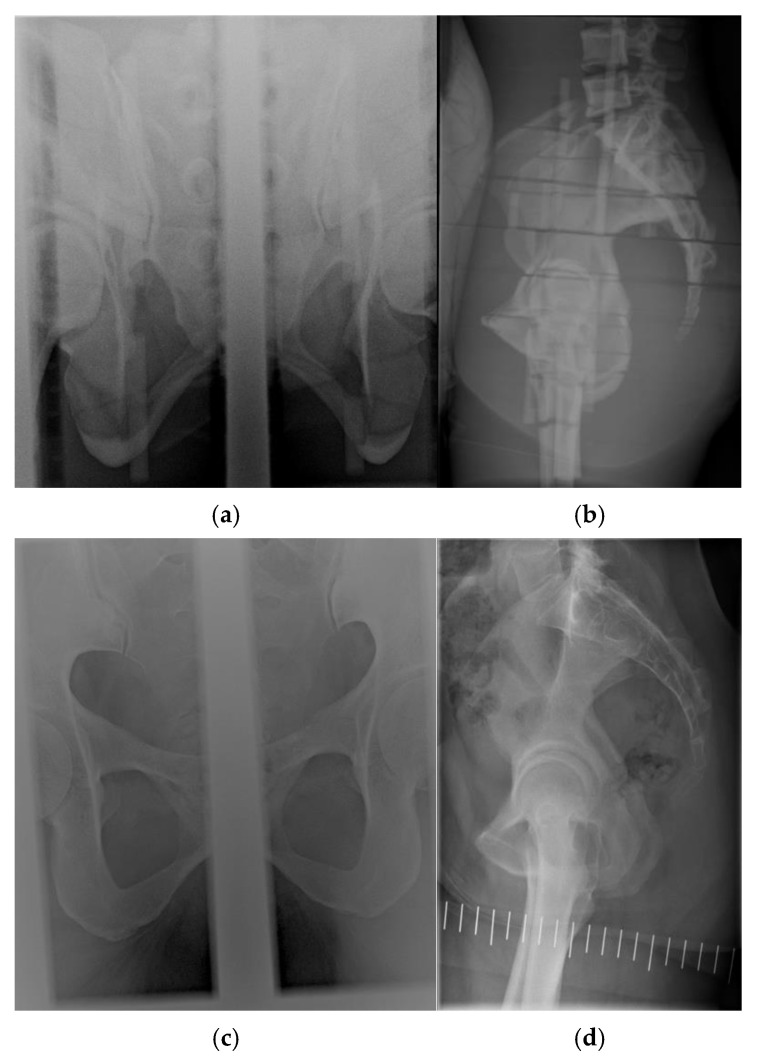
Pelvimetry 2D images using the female Alderson phantom, consisting of two AP bedside images that were (**a**) matched on the same CR receptor using computed radiography (CR) by acquiring two individual exposures and (**b**) acquired using digital radiography (DR), and an example of patient images acquired in the same way, (**c**) and (**d**), respectively. The white vertical lines represent radiographic reference markers used during pelvimetry acquisition.

The acquisition parameters applied to the phantom were identical to those used clinically (Figure 2). During the conventional X-ray examination, frontal anterior–posterior (AP) and lateral projections were obtained. In the AP view, the X-ray tube was tilted 22° caudally. A computed radiography (CR) cassette was placed beneath the pelvis and centered at the midline. The tube or examination table was then shifted approximately 7 cm to the right for the first exposure (66 kV, 20 mAs) and then repositioned to the midline before being moved 7 cm to the left for the second exposure. Each image displayed approximately half of the pelvis (Figure 2a,c; collimation ~7 cm × 17 cm). Two exposures on the CR plate were used to minimize magnification effects caused by beam divergence. The lateral projection included the sacrum and symphysis pubis and was centered on the symphysis and laterally over the trochanter major (Figure 2b,d; collimation approximately 24 cm × 15.5 cm).

Pelvimetry radiographs of the phantom were acquired using a Philips Optimus 50 X-ray DR system (Philips Medical Systems, Best, The Netherlands) [9], with CR cassettes (Fujifilm Medical Systems, Tokyo, Japan) for AP exposures and a DR detector for lateral projections (Table 1). AP exposures were performed with fixed parameters, whereas automatic exposure control adjusted the tube load for the lateral DR projection. Dose-area products (DAPs) were recorded for effective-dose calculations and monitored using a DAP meter calibrated annually. Neither phantom images nor patient images from conventional examinations were included in the image quality evaluation; only radiation dose measurements were evaluated for conventional pelvimetry.

#### 2.3.2. Low-Dose CT Pelvimetry Protocol

CT pelvimetry was performed using a low-dose helical protocol on a Canon Aquilion Prime CT scanner (Canon Medical Systems Corporation, Otawara, Japan). The protocol included a frontal scout image (80 kV, 10 mA) followed by a helical acquisition (100 kV, 7 mAs), resulting in a volume CT dose index (CTDI_vol_) of 0.5 mGy (Table 2).

In CT pelvimetry examinations, patients were scanned in supine position. Pelvic circumference was either measured manually using a tape measure or derived from CT images at the acetabular level to evaluate potential associations between patient size and image quality.

#### 2.3.3. Monte Carlo-Based Effective Dose Estimates for a Standard-Sized Female

Two dedicated Monte Carlo (MC) programs were used to calculate effective dose: CT-Expo (version 2.5, SASCRAD, Buchholz, Germany) for scanner-specific CT examinations, including scout images, and PCXMC (version 2.0, Finnish Radiation and Nuclear Safety Authority, Helsinki, Finland) for conventional radiography [17,18].

For both pelvimetry modalities, effective dose was calculated for an adult female of standard size (160 cm/60 kg). PCXMC employs a sex-averaged phantom model; therefore, contributions to the male gonads were excluded by subtracting the dose equivalents to the testes and prostate. Female gonadal tissue weighting factors (Wt) were then adjusted to sum to unity, since the remaining dose represents an arithmetic average of the male and female contributions [17,18].

In addition to the mAs values, the acquisition parameters in Table 1 and Table 2 were used to calculate effective doses according to tissue weighting factors defined by the International Commission on Radiological Protection in (ICRP 103) [19].

#### 2.3.4. Effective Dose Estimates Based on Patient Data

Dose Area Product (DAP) values from consecutive conventional pelvimetry examinations conducted over a three-year period (30 June 2014, to 4 May 2017) and Dose Length Product (DLP) from the first 14 CT patient exams were compiled using DOSESTAT^®^ software (Viximed AB, Uppsala, Sweden; version 3.0). Data were retrieved from the Picture Archiving and Communication System (PACS; Sectra Workstation IDS7, Sectra AB, Linköping, Sweden, version 18.1).

The search encompassed multiple hospitals within the region and included various conventional DR X-ray systems: Philips Optimus 50 X-ray (Philips Medical Systems, Best, The Netherlands), Fujifilm’s FDR Acselerate (Fujifilm Medical Systems, Tokyo, Japan), Adora DRFi (Mediel AB, Lomma, Sweden), and Siemens Polydoros DR (Siemens Healthcare GmbH, Erlangen, Germany).

This dataset included examinations with and without retakes to account for the total dose contributions from frontal and lateral projections. Effective dose was estimated from average DAP and DLP values using pelvis-specific conversion coefficients: 0.00014 mSv/mGy·cm^2^ for conventional radiographic pelvimetry [25] and 0.0143 mSv/mGy·cm for CT pelvimetry [26].

#### 2.3.5. Image Quality Evaluation of the CT Pelvimetry Examinations

Two radiologists with 20 and 11 years of general radiology experience (including 12 and 5 years, respectively, in pelvimetry interpretation) independently evaluated the image quality of relevant anatomy in CT volumes from 14 consecutive patients examined using the protocol described in Table 2.

Images were reviewed on calibrated 2-megapixel medical monitors. Window and level settings were adjustable to preference. Image quality was assessed using a four-point scale:

0 = insufficient,
1 = barely sufficient,
2 = acceptable,
3 = clearly adequate for diagnostic assessment.

Evaluation focused on anatomical landmarks required for pelvimetric measurements, including the transverse diameter of the pelvic inlet, interspinous and intertuberous distances, sagittal pelvic outlet diameter, and obstetric conjugate, involving visualization of the hip bones, sacrum, and coccyx. Readers were instructed to assign a single overall rating per case when image quality was consistent across all structures. If one or more anatomical structures exhibited reduced visibility, the lower rating was applied, and the quality of other structures was noted. Image quality ratings were analyzed in relation to patients’ pelvic circumferences. Interobserver agreement was assessed using Cohen’s kappa statistic (MedCalc Software, version 23.3.7) [27,28]. In addition, CT examinations were reviewed to confirm completion of clinical measurements and to determine whether any cases required recall due to insufficient image quality.

## 3. Results

### 3.1. Phantom Dose Assessment Using TLDs

**Figure 3:**
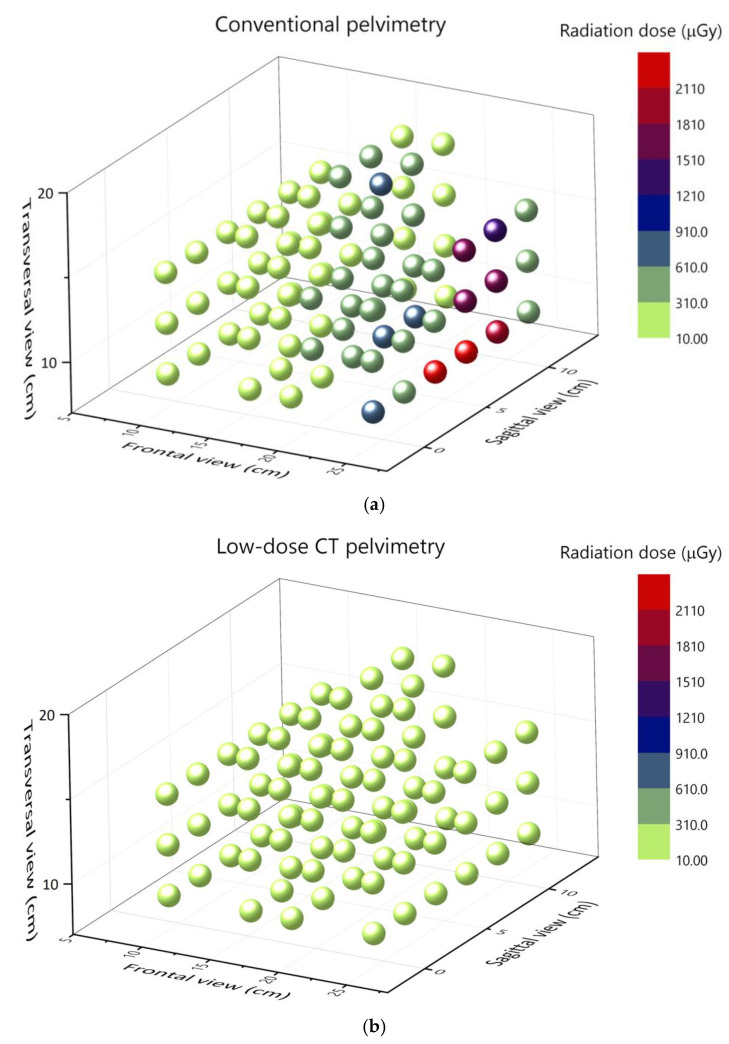
The absorbed dose distribution (mGy) within the anthropomorphic phantom after (**a**) the conventional pelvimetry examination using radiography and (**b**) low-dose CT pelvimetry, which includes one scout image and a helical scan. Each circle represents a TLD measurement at a specific location in the phantom. The dose at each point is indicated by color, with the measurement (mGy) shown on the color bar on the right.

The CT protocol produced an average absorbed pelvic dose (mGy) approximately half that of conventional radiographic pelvimetry (Table 3), with fetal doses approximately 40% lower. These fetal dose estimates were derived from standardized single acquisitions and therefore do not account for the additional radiation exposure that may result from retakes in clinical conventional practice. Inclusion of retakes would be expected to further increase the fetal dose in conventional pelvimetry and thereby widen the relative dose difference between modalities. The average absorbed dose across all TLD positions (slabs 26–31) during CT was 0.18 mGy, compared with 0.39 mGy during conventional pelvimetry. CT demonstrated a more uniform dose distribution across slabs (Figure 3), as reflected by lower standard deviation values (Table 3) and the individual TLD measurements illustrated in Figure 3. In contrast, conventional pelvimetry exhibited marked dose heterogeneity, with peak values reaching 2.3 mGy.

The total DAP recorded during phantom conventional imaging was 2344 mGy·cm^2^ (166 and 159 mGy·cm^2^ for the left and right frontal projections, respectively, and 2019 mGy·cm^2^ for the lateral projection). The CT dose indicator (DLP) for the helical scan was 11 mGy·cm.

### 3.2. Effective Dose Assessment

#### 3.2.1. Anthropomorphic Phantom Data

Effective dose calculations based on the actual acquisition parameters using PCXMC and CT-expo for a female (160 cm/60 kg) are presented in Table 4. Conventional pelvimetry yielded an overall effective dose more than twice that of low-dose CT pelvimetry (0.357 mSv vs. 0.164 mSv). The effective dose from the right frontal projection was nearly twice that of the left frontal image, likely reflecting greater inclusion of more radiosensitive tissues within the beam field. The lateral projection contributed substantially to total exposure, with an effective dose 2.8 times higher than the combined frontal projections.

#### 3.2.2. Effective Dose Data Based on Patients for Conventional Pelvimetry and Initial Helical CT Pelvimetry

**Figure 4:**
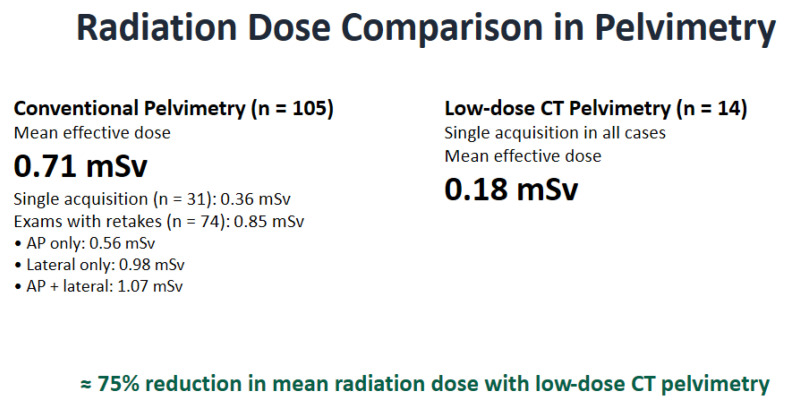
Comparison of effective radiation dose between conventional radiographic pelvimetry and low-dose CT (LD-CT) pelvimetry. Conventional pelvimetry (*n* = 105) was associated with a higher mean effective dose (0.71 mSv), largely due to image retakes in most examinations. Retakes most often involved lateral or combined anteroposterior (AP) and lateral views, thereby increasing radiation exposure. In contrast, LD-CT pelvimetry (*n* = 14) required a single acquisition in all cases and was associated with a substantially lower mean effective dose (0.18 mSv), corresponding to an approximate 75% reduction in radiation dose compared with conventional techniques.

The database search identified 105 patient examinations performed with conventional pelvimetry. Retakes occurred in 70.5% of cases (Table 5). There was close agreement between phantom-based effective dose estimate and the patient-derived values for examinations without retakes (0.357 vs. 0.362 mSv), supporting the validity of the modelling approach. When retakes were included, the mean effective dose for conventional pelvimetry increased to 0.71 mSv, approximately double that of cases without retakes. Subgroup analysis showed that lateral-view retakes contributed disproportionately to total dose, with an average effective dose of 1.09 mSv in those cases. These accounted for approximately 29.5% of all examinations. In contrast, no retakes or recalls were required in the CT cohort. The average effective dose was 0.18 mSv (Figure 4).

### 3.3. Results on Image Quality of Low-Dose CT Pelvimetry Examinations

**Figure 5:**
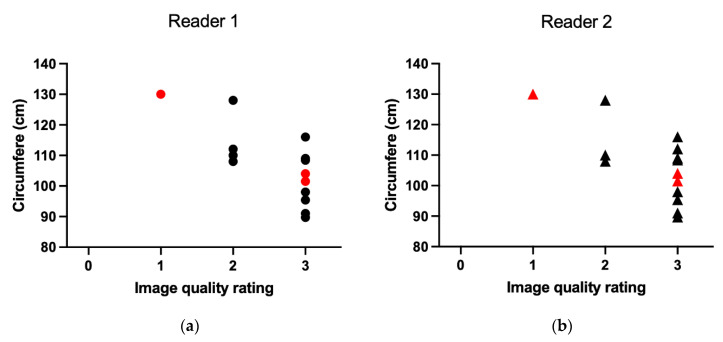
The relationship between patients’ circumferences and image quality, as interpreted by two radiologists (**a**,**b**). The red points represent data from pregnant women in the study sample.

The study population included women aged 22–35 (mean age of 26.9 years). Pelvic circumferences ranged from 89.7 cm to 130 cm (mean 106.7 cm). Three patients were pregnant; the remaining patients were referred primarily due to obstetric concerns related to previous pregnancies. For all examinations, both radiologists agreed that coronal-plane visualization of the pelvic inlet, interspinous, and intertuberous distances was clearly adequate. Variability in sagittal-plane image quality, particularly regarding visualization of the sacrum and coccyx, appeared to be associated with increased patient circumference (Figure 5). However, this observation is based on a limited sample-size.

**Figure 6:**
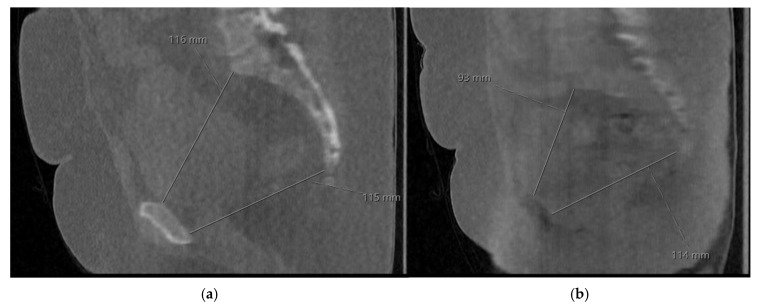
Examples of CT pelvimetry cases with (**a**) clearly sufficient image quality and (**b**) low image quality, making it difficult to define the sacrum and coccyx (tailbone). The measures shown are the medial obstetric conjugate (inlet) and the sagittal pelvic outlet diameter.

Image quality was rated as clearly adequate (score 3) in nine and ten cases by readers 1 and 2, respectively. Ratings of acceptable (score 2) were assigned in four and three cases and one case was rated as barely sufficient (score 1). All cases were rated sufficient (score 1 or higher). Reduced visualization of the sacrum and coccyx in sagittal reconstructions accounted for lower ratings in certain cases, whereas larger osseous structures remained clearly visible. Interobserver agreement was high (Cohen’s ĸ = 0.85; observed agreement 92.9%), indicating almost perfect agreement. Figure 6a presents an example case rated clearly adequate, while Figure 6b shows the only case rated barely sufficient for the visibility of the coccyx. Follow-up confirmed that all CT examinations allowed successful pelvimetric measurements and that no patient required recall due to insufficient image quality.

## 4. Discussion

This study demonstrates that CT pelvimetry can be performed at substantially lower radiation doses than conventional radiographic pelvimetry while maintaining adequate image quality for clinical measurements. With a low-dose helical CT protocol, the average absorbed pelvic dose was approximately 50% lower than that of conventional pelvimetry, with an estimated 40% reduction in fetal dose. Importantly, the fetal dose comparison was based on standardized single-exposure phantom measurements and did not incorporate the effect of retakes observed in clinical conventional pelvimetry. Given the high frequency of retakes in conventional examinations, inclusion of these additional exposures would likely have resulted in an even greater relative reduction in fetal dose in favor of CT. In addition, CT pelvimetry provided a more uniform dose distribution, whereas conventional pelvimetry exhibited pronounced dose heterogeneity, primarily due to the lateral projection.

When effective-dose estimates were considered, CT pelvimetry delivered less than half the effective dose of conventional pelvimetry, based on phantom-derived acquisition parameters. Importantly, retrospective patient data showed that retakes were common in conventional pelvimetry, occurring in more than 70% of examinations. Including retakes approximately doubled the effective dose, and lateral-view retakes contributed disproportionately to the total exposure. In contrast, no retakes or recalls were observed for CT pelvimetry, resulting in a substantially lower overall radiation burden.

The higher dose associated with conventional pelvimetry is largely attributable to the lateral projection, which relies on automatic exposure control and is sensitive to patient size and tissue density. This likely explains both the higher effective dose and the wide variability in phantom measurements. In CT pelvimetry, dose is distributed across a volumetric acquisition, resulting in greater dose homogeneity and reduced peak exposures.

From an image quality perspective, all CT examinations were considered diagnostically sufficient for pelvimetric measurements. Slight image degradation in the sagittal plane was observed in patients with larger pelvic circumferences, primarily affecting visualization of the sacrum and coccyx. However, minor measurement deviations on the order of millimeters are unlikely to have a meaningful impact on the overall pelvimetric assessment, and no examination was found to be insufficient regarding relevant anatomy or to require recall. Further protocol optimization remains feasible, for example by adapting tube current to patient size within predefined safety limits (i.e., mA constraints). Such adjustments may allow additional dose reduction in smaller patients while maintaining adequate image quality. Moreover, the use of volume scanning techiques could further reduce radiation dose (~15–20%) by reducing overscanning. Overall, there appears to be a substantial margin for dose adjustment in CT pelvimetry without reaching the higher exposure levels observed with conventional radiographic techniques (Table 5).

Several limitations should be acknowledged. The clinical image quality assessment was based on a limited patient cohort, and fetal dose estimates were derived from uterine TLD placement in an anthropomorphic phantom rather than direct in vivo dosimetry. Although this approach is widely used and provides standardized comparison, it cannot fully account for anatomical variation during pregnancy. The CT protocol was implemented on a single scanner platform, and dose efficiency may vary depending on vendor-specific hardware and reconstruction algorithms. Therefore, extrapolation of exposure settings to other CT systems should be made with caution. In addition, although image quality was evaluated, the study did not include a direct comparison of pelvimetric measurement accuracy against an independent reference standard. Nevertheless, the combined use of physical dosimetry, Monte Carlo–based modeling, and retrospective patient dose data provides a consistent and conservative framework for comparing the two imaging modalities.

Improved diagnostic capability offers several potential advantages in pelvimetric assessment. While traditional measurement methods rely on projection-based techniques, CT enables multiplanar and three-dimensional reconstructions that may enhance geometric accuracy and reproducibility. Advanced post-processing tools, including multiplanar reformations (MPRs), maximum intensity projections (MIPs), three-dimensional reconstructions, and emerging AI-assisted methods, may further improve measurement precision and workflow efficiency. Continued research is warranted to refine and standardize CT-based pelvimetric measurement techniques and to establish consensus regarding optimal acquisition and reconstruction parameters [29].

## 5. Conclusions

Optimized low-dose CT pelvimetry significantly reduces radiation dose compared with conventional radiographic pelvimetry while maintaining reliable diagnostic image quality. Taking into account the frequent retakes in conventional imaging, the reduction in effective dose becomes particularly pronounced. In addition to improved dose efficiency, CT provides more homogeneous radiation distribution and overcomes geometric limitations inherent to projection-based techniques.

Taken together, these findings support the transition to optimized low-dose CT pelvimetry as a safer, more reproducible, and dose-efficient strategy for clinical pelvimetric assessment.

## Figures and Tables

**Table 1:** The parameters used for conventional pelvimetry during phantom imaging. These settings are identical to those used clinically. Still, when imaging patients, the mAs value may differ in the lateral projection due to the automatic exposure control, which depends on patient thickness/density. Al = aluminum filtration.

	Anterior–Posterior Image		Lateral Image
Tube Voltage (kV)	66	66	90
Exposure time product (mAs)	20	20	49
Source-to-image distance (cm)	100	100	100
Added filtration	2 mm Al	2 mm Al	2 mm Al
Grid	-	-	Yes

**Table 2:** Parameters utilized for CT pelvimetry scanning.

Parameters	Pelvimetry-CT *^,£^
Collimation (mm)	80 × 0.5
Scanning mode	Helical
Slice thickness (mm)	5.0
Pitch	0.813
Rotation time (s)	0.35
Tube voltage (kVp)	100
Tube current time product (mAs)	7
Orientation	Head first

* Iterative reconstruction (AIDR-3D). ^£^ Three patients were examined with slightly modified settings from those presented; two cases used a mAs value of 8, and one case used 10 mAs.

**Table 3:** The average, standard deviation, and maximum absorbed doses for CT and conventional radiography pelvimetry are presented for the fetus and all selected slabs.

Slab (No.)	Average (mGy)		Standard Deviation		Maximum (mGy)	
	Conventional	CT	Conventional	CT	Conventional	CT
26	0.178	0.190	0.185	0.046	0.620	0.265
27	0.197	0.193	0.189	0.028	0.607	0.256
28	0.423	0.183	0.545	0.017	2.252	0.217
29	0.722	0.180	0.639	0.023	2.231	0.221
30	0.651	0.175	0.564	0.019	1.976	0.213
31	0.182	0.183	0.151	0.017	0.533	0.208
Fetus	0.368	0.215	0.097	0.008	0.523	0.148
Average:	0.392	0.172	0.487	0.050	2.252	0.265

**Table 4:** The effective dose derived from both phantom- and patient-based data for conventional pelvimetry and for CT pelvimetry.

Effective Dose (mSv)			
Conventional Pelvimetry		CT Pelvimetry ^£^	
Left frontal image	0.033	Frontal scout *	0.0069
Right frontal image	0.062	Helical scan	0.157
Lateral image	0.262		
Total:	0.357		0.164

*^£^* The effective dose of the helical scan was estimated at 0.12 mSv for CT pelvimetry using a Canon Aquilion One Genesis CT scanner. * The frontal scout was acquired at 80 kV and 10 mA, yielding a DLP of 0.34 mGy·cm.

**Table 5:** The number of examinations, average dose-area product (DAP) values, and the resulting effective dose are presented separately for cases with and without retakes. Cases with retakes are further divided into those with retakes performed in the AP view(s) only (range: 1 to 5 retakes, avg. = 2.9), the lateral view(s) only (range: 1 to 4 retakes, avg. 1.8), and both views (2 to 6 retakes, avg. 3.0). In addition, average estimates are presented for the 14 initial patients examined with low-dose helical CT pelvimetry.

Conventional Pelvimetry *			
	Number of Examinations	Average DAP (mGy·cm^2^)	Effective Dose (mSv)
All cases	105	5054	0.71
No retakes	31	2590	0.36
Retakes	74	6047	0.85
AP view(s), only	25	4026	0.56
Lateral view(s), only	22	7027	0.98
Both views retaken	27	7665	1.07
CT pelvimetry *			
	Number of examinations	Average DLP (mGy·cm)	Effective dose (mSv)
Helical CT	14	12.8	0.183

* Estimates are based on sex-average phantom models.

## Data Availability

The raw data supporting the conclusions of this article will be made available by the authors on request.
